# Regulation of NF-κB Activation through a Novel PI-3K-Independent and PKA/Akt-Dependent Pathway in Human Umbilical Vein Endothelial Cells

**DOI:** 10.1371/journal.pone.0046528

**Published:** 2012-10-05

**Authors:** Sakshi Balwani, Rituparna Chaudhuri, Debkumar Nandi, Parasuraman Jaisankar, Anurag Agrawal, Balaram Ghosh

**Affiliations:** 1 Molecular Immunogenetics Laboratory, CSIR-Institute of Genomics and Integrative Biology, Delhi, India; 2 Department of Medicinal Chemistry, CSIR-Indian Institute of Chemical Biology, Jadavpur, Kolkata, India; Ottawa Hospital Research Institute, Canada

## Abstract

The transcription factor NF-κB regulates numerous inflammatory diseases, and proteins involved in the NF-κB-activating signaling pathway are important therapeutic targets. In human umbilical vein endothelial cells (HUVECs), TNF-α-induced IκBα degradation and p65/RelA phosphorylation regulate NF-κB activation. These are mediated by IKKs (IκB kinases) viz. IKKα, β and γ which receive activating signals from upstream kinases such as Akt. Akt is known to be positively regulated by PI-3K (phosphoinositide-3-kinase) and differentially regulated via Protein kinase A (PKA) in various cell types. However, the involvement of PKA/Akt cross talk in regulating NF-κB in HUVECs has not been explored yet. Here, we examined the involvement of PKA/Akt cross-talk in HUVECs using a novel compound, 2-methyl-pyran-4-one-3-O-β-D-2′,3′,4′,6′-tetra-O-acetyl glucopyranoside (MPTAG). We observed that MPTAG does not directly inhibit IKK-β but prevents TNF-α-induced activation of IKK-β by blocking its association with Akt and thereby inhibits NF-κB activation. Interestingly, our results also revealed that inhibitory effect of MPTAG on Akt and NF-κB activation was unaffected by wortmannin, and was completely abolished by H-89 treatment in these cells. Thus, MPTAG-mediated inhibition of TNF-α-induced Akt activation was independent of PI-3K and dependent on PKA. Most importantly, MPTAG restores the otherwise repressed activity of PKA and inhibits the TNF-α-induced Akt phosphorylation at both Thr308 and Ser473 residues. Thus, we demonstrate for the first time the involvement of PKA/Akt cross talk in NF-κB activation in HUVECs. Also, MPTAG could be useful as a lead molecule for developing potent therapeutic molecules for diseases where NF-κB activation plays a key role.

## Introduction

Nuclear transcription factor-κB (NF-κB) plays a central role in inflammation and apoptosis through diverse signaling cascades. Up-regulation of cell adhesion molecules by NF-κB on endothelial cells is a critical step which alters the adhesive property of vasculature and causes uncontrolled infiltration of leukocytes into the inflamed tissue. Pharmacological inhibitors of NF-κB pathway in endothelial cells have potential therapeutic value in treating inflammatory diseases and cancers [1], [2]. NF-κB has been detected in most cell types and consists of a p50/p65 heterodimer, which is retained in the cytoplasm by the masking of nuclear localization sequence by IκBα, the inhibitor of NF-κB [3]. Induction of human umbilical vein endothelial cells (HUVECs) with proinflammatory stimuli such as TNF-α, IL-1β and bacterial lipopolysaccharide (LPS) leads to IκBα phosphorylation, ubiquitination, and subsequent degradation resulting in the release of p50/p65 heterodimer [4]. The heterodimers of NF-κB migrate into the nucleus and activate the expression of numerous target genes that are important for inflammatory and immune responses as well as other functions, such as the regulation of apoptosis [5] and cell proliferation [6]. The inducible phosphorylation of IκBα is mediated by IκB kinases (IKKs) [7]. IKKs comprises of three subunits: IKKα/IKK1 and IKKβ/IKK2, that are catalytic [8] while the third, called IKKγ or NF-κB essential modulator (NEMO), is regulatory [9]. In human umbilical vein endothelial cells (HUVECs), IKKs are themselves direct downstream targets for various IKK-activating kinases such as Akt and TAK1 [10]–[12]. In addition, MAP kinases, such as p38 and ERK are activated upon TNF-α stimulation and are known to be associated with NF-κB activation in various cell types including HUVECs [13].

Akt is activated by TNF-α through the phosphoinositide-3-kinase (PI-3K) pathway in various cells including HUVECs. In addition to its anti-apoptotic functions, Akt can stimulate signaling pathways that upregulate the activity of the transcription factor NF-κB. Wortmannin (a specific PI-3K inhibitor) or dominant-negative PI-3K or kinase-dead Akt inhibits the TNF-α-mediated NF-κB activation in these and other cells [14]–[19]. Moreover, Akt is known to be differentially regulated via Protein kinase A (PKA) in various cell types [20]–[24]. This PKA/Akt axis is poorly explored in NF-κB activation, compared to the classical PI-3K/Akt pathway, and offers opportunity for drug discovery.

**Figure 1:**
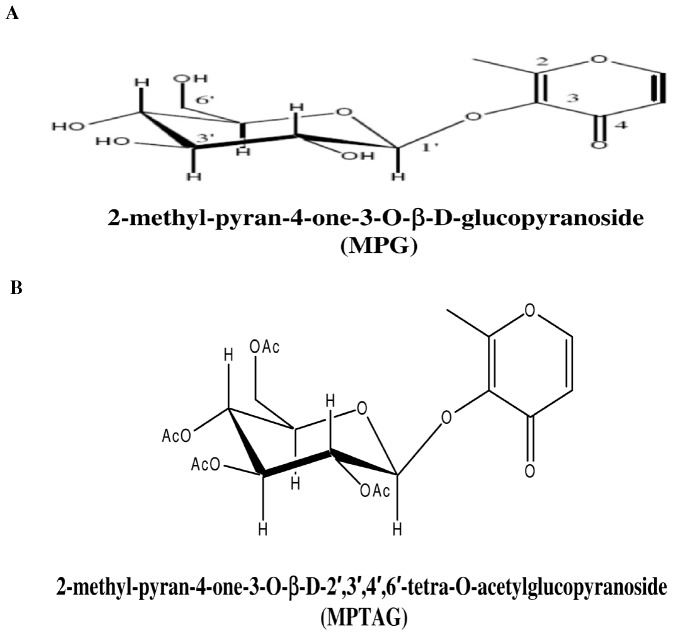
The structures of MPG, a novel compound isolated and purified from *P. granatum* leaves (A) and MPTAG, the most active laboratory synthesized derivative of MPG (B).

The present report attempts to address the yet unexplored mechanism of PKA/Akt-dependent phosphorylation and activation of NF-κB in HUVECs. Numerous compounds, synthetic and plant-derived, have been demonstrated to inhibit NF-κB activation either through direct PI3K inhibition or specific IKK inhibition or proteasome pathway blockade [25]–[27]. However, small molecules that inhibit NF-κB activation through modulation of the PKA/Akt axis in TNF-α-stimulated HUVECs have not been studied previously. Earlier, we identified 2-methyl-pyran-4-one-3-O-β-D-glucopyranoside (MPG; Figure 1A), a novel compound isolated from the leaves of *Punica granatum*, which inhibited the TNF-α-induced cell adhesion molecules expression [28].

In this paper, using a novel derivative of MPG, 2-methyl-pyran-4-one-3-O-β-D-2′,3′,4′,6′-tetra-O-acetyl glucopyranoside (MPTAG; Figure 1B), we demonstrate for the first time that MPTAG could inhibit the activation of NF-κB through a PI3K-independent but PKA/Akt-dependent pathway in TNF-α-stimulated HUVECs. This represents a novel mechanism of NF-κB regulation and its implication in developing novel anti-inflammatory agents is discussed.

## Results

### Synthesis of derivatives of MPG

Earlier, we demonstrated that MPG (Figure 1A) inhibited the TNF-α-induced cell adhesion molecules (ICAM-1, VCAM-1 and E-selectin) expression and adhesion of neutrophils to the endothelium monolayer in a dose-dependent manner [28]. Encouraged with these results, we synthesized derivatives of MPG in sufficient amounts in the laboratory (Figure S1 and Table S1). The derivatives were tested for their solubility, cytotoxicity and ability to inhibit the TNF-α-induced expression of ICAM-1 (intercellular cell adhesion molecule-1), VCAM-1 (vascular cell adhesion molecule-1) and E-selectin as well as neutrophil adhesion on human endothelial cells. The results revealed that 2-methyl-pyran-4-one-3-O-β-D-2′,3′,4′,6′-tetra-O-acetyl glucopyranoside (MPTAG; Figure 1B) was the most active derivative (Table S2). We also found that it was completely water soluble and less cytotoxic as compared to parent compound, MPG and the other two derivatives (Tables S2 and S3). Thus, MPTAG was taken up for further experiments to unravel its molecular mechanism of action in HUVECs.

### MPTAG inhibits TNF-α-induced NF-κB activation in endothelial cells

**Figure 2:**
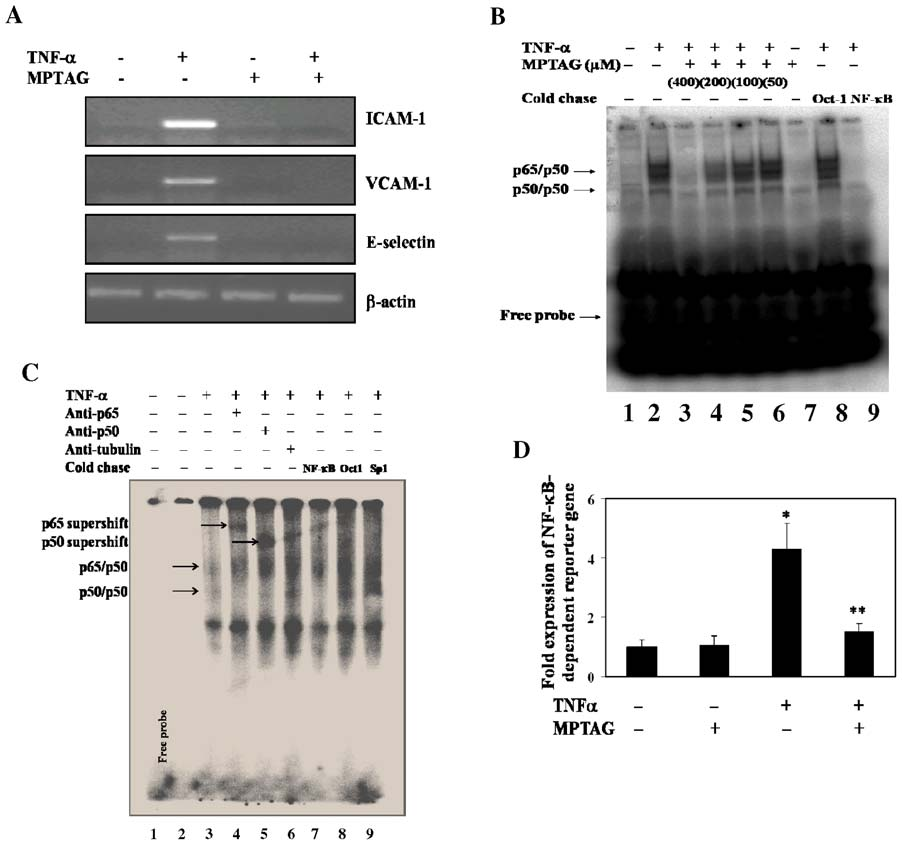
MPTAG prevents the TNF-α-induced NF-κB transcription and activation in human endothelial cells. (A) The cells were pretreated with or without 400 µM of MPG before induction with TNF-α (10 ng/ml) for 4 h. The total RNA of the cells was isolated and analysed by RT-PCR. The intensity of transcripts was normalized with that of β-Actin levels expressed under similar conditions. (B) The cells were pretreated with MPTAG at varying concentrations and then induced with TNF-α. The cytoplasmic (CE) and nuclear (NE) extracts were prepared from untreated and MPTAG-treated TNF-α-stimulated cells (see “Methods”). The nuclear extracts were analyzed for NF-κB activation by EMSA. (C) The nuclear extracts from unstimulated or TNF-α-stimulated HUVECs were incubated with the indicated antibodies and analyzed for NF-κB activation by EMSA (see “Methods”). (D) The cells were transiently transfected by electroporation with a NF-κB-containing luciferase reporter gene followed by treatment with 400 µM MPTAG and TNF-α stimulation. The supernatants after cell lysis were assayed for luciferase activity. The mean value for cells treated with no MPTAG and no TNF-α was set to 1, and -fold differences were determined by comparing values against this set value. *p<0.005 vs. uninduced cells; **p<0.01 vs. TNF-α-induced cells, statistical difference was set at p<0.05.

Further experiments revealed that MPTAG exhibited maximum inhibition of ICAM-1, VCAM-1 and E-selectin, when added 2–4 h prior to TNF-α stimulation to human endothelial cells (data not shown). As the promoter regions of the genes encoding cell adhesion molecules (CAMs) contain binding site(s) for NF-κB, we measured the transcript levels of these genes by RT-PCR (Figure 2A). The results showed that in uninduced cells treated with or without MPTAG, the levels of ICAM-1, VCAM-1 and E-selectin mRNAs were very low. In contrast, upon stimulation of cells with TNF-α, there was an upregulation in ICAM-1, VCAM-1 and E-selectin mRNA levels. However, pretreatment of endothelial cells with MPTAG significantly reduced the induced mRNA levels of ICAM-1, VCAM-1 and E-selectin (Figure 2A).

Since NF-κB is a key transcription factor involved in the expression of CAMs in endothelial cells, we determined its activation in MPTAG-pretreated cells by EMSA (Figure 2B). There was a low level of NF-κB activation in unstimulated cells in absence and presence of MPTAG (lanes 1 and 7). Upon stimulation with TNF-α, there was an increased NF-κB DNA-binding activity as expected (lane 2). However, MPTAG pretreatment caused a substantial reduction in this activity in a dose-dependent manner (lanes 3–6). MPTAG pretreatment resulted in almost complete inhibition of NF-κB DNA-binding activity at a concentration of 400 µM (lane 3). The specificity of the NF-κB DNA complex was confirmed by incubation of the nuclear extract proteins with an excess of unlabeled NF-κB oligonucleotide that inhibited the complex formation (lane 9) whereas competition with an excess of an irrelevant oligonucleotide, Oct-1, did not inhibit the complex (lane 8). The specificity was further confirmed by NF-κB supershift assay (Figure 2C). The nuclear extracts prepared from unstimulated (lane 2) and TNF-α-stimulated cells (lanes 3–9) were incubated with antibodies against the p65 subunit (lane 4) or p50 (lane 5) of NF-κB. The antibodies shifted the p65 and p50 bands to higher molecular mass (lanes 4 and 5, respectively), suggesting that the TNF-α-activated NF-κB complex consisted of both p50 and p65 subunits. On the other hand, an irrelevant anti-α-tubulin (lane 6) or oligonucleotide specific to Oct1 (lane 8) or Sp1 (lane 9) had no effect. Thus, MPTAG pretreatment significantly inhibited TNF-α-induced activation of NF-κB in endothelial cells.

The inhibition of NF-κB activation by MPTAG was further assessed by transfection of NF-κB-regulated luciferase reporter construct in endothelial cells as described in “*Methods*”. For this, endothelial cells were transiently transfected with the luciferase vector, preincubated with MPTAG and stimulated with TNF-α. We observed that luciferase activity was significantly inhibited by MPTAG (Figure 2D).

### MPTAG inhibits TNF-α-induced NF-κB translocation in endothelial cells

**Figure 3:**
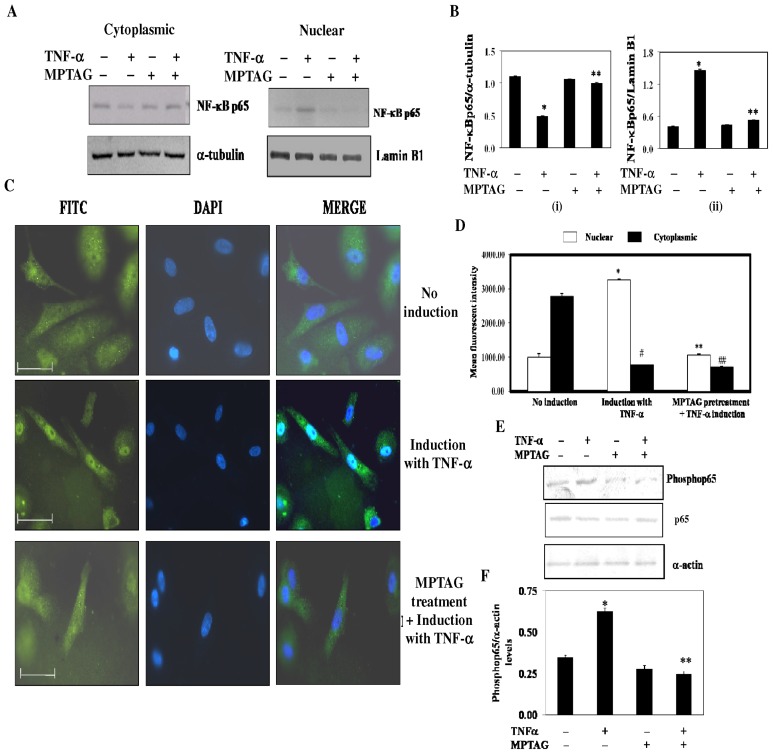
MPTAG prevents the TNF-α-induced NF-κB translocation in human endothelial cells. (A) The cells were pretreated with MPTAG (400 µM) and induced with TNF-α. The cytoplasmic (CE) and nuclear (NE) extracts were prepared and processed for western blot analysis. (B) The intensity of bands were densitometrically scanned and normalized with that of α-tubulin and Lamin B1 in CE and NE extracts, respectively. The values presented are mean ± SEM. *p<0.005 vs. uninduced cells; **p<0.01 vs. TNF-α-induced cells, statistical significance was set at p<0.05. (C–D) The cells were treated and induced as described in ‘A’ and subjected to immunocytochemical analysis using anti-NF-κBp65 and FITC-labeled anti-rabbit antibodies. DAPI was used for staining the nucleus. The mean intensity levels of NF-κB conjugated to FITC, both in the cytoplasm and nucleus, were quantitated and plotted as mean intensity ± SEM. *,#p<0.05 vs. uninduced cells; **,##p<0.05 vs. TNF-α-induced cells, statistical difference was set at p<0.05. The scale bars represent 50 µm (E–F) **MPTAG inhibits TNF-α-induced p65 phosphorylation in endothelial cells.** The cells were treated and induced followed by western blot analysis as stated in ‘A’. The intensity of bands were densitometrically scanned and normalized with that of α-actin levels. The values presented are mean ± SEM. *p<0.05 vs. uninduced cells; **p<0.02 vs. TNF-α-induced cells, statistical difference was set at p<0.05.

Since NF-κB activation requires the translocation of its p65 subunit to the nucleus, we measured the levels of p65 in the cytoplasm and in the nucleus in MPTAG-pretreated cells. It was observed that there were high levels of p65 in the cytoplasm of unstimulated cells in absence and presence of MPTAG and the levels were found to be very low in the nucleus (Figures 3A and 3B). Upon stimulation of cells with TNF-α, as expected, the levels of p65 in the cytoplasm were decreased with a concomitant increase in the nucleus (Figures 3A and 3B). MPTAG pretreated TNF-α stimulated cells had higher cytoplasmic levels of p65 than cells that were only TNF-α stimulated. There was a concomitant decrease in the nuclear p65 levels (Figures 3A and 3B). These results indicated that MPTAG prevented the translocation of NF-κBp65 subunit into the nucleus of endothelial cells. This was additionally confirmed by immunocytochemical analysis (Figures 3C and 3D).

It is reported that TNF-α stimulation results in the phosphorylation of p65 subunit of NF-κB in the cytoplasm, which is required for its nuclear translocation and transcriptional activity [29]. Therefore, we determined the effect of MPTAG on p65 phosphorylation in the cytoplasm. We observed that in cells pretreated with MPTAG, TNF-α was unable to induce p65 phosphorylation (Figures 3E and 3F) indicating that MPTAG inhibits p65 phosphorylation.

### MPTAG prevents TNF-α-induced IκBα degradation by inhibiting the IKK-β activation

**Figure 4:**
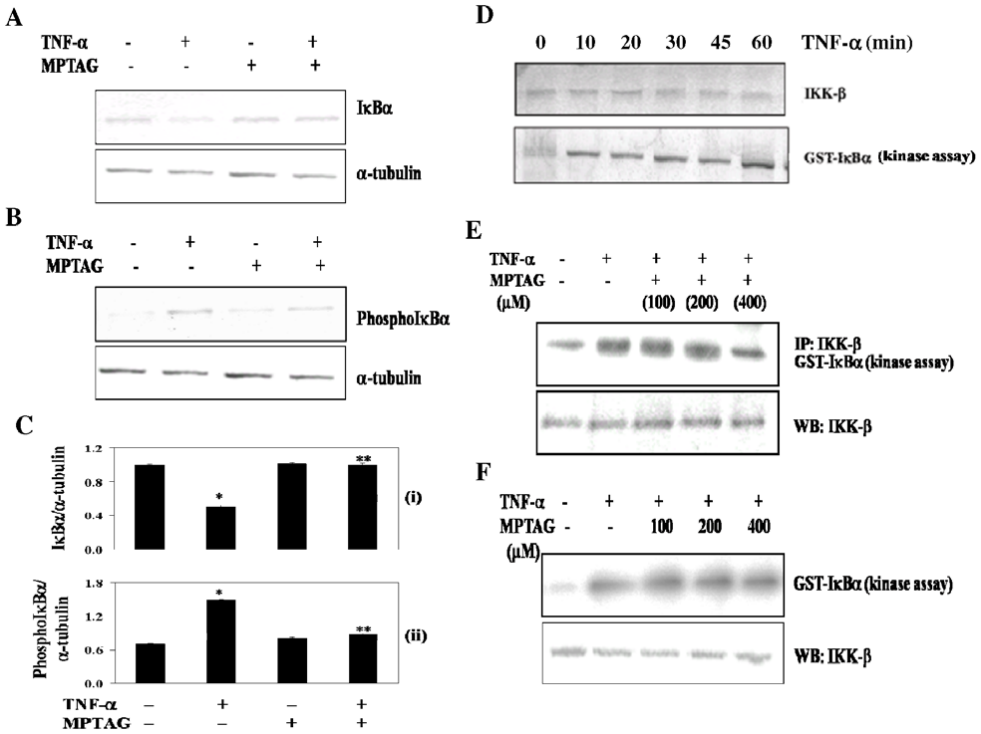
MPTAG prevents TNF-α-induced IκBα degradation by inhibiting the activation of IKK-β. (A–B) The cells were treated with MPTAG (400 µM) followed by induction with TNFα for 30 mins and total cell extracts were processed for western blot analysis using antibodies against IκBα, phosphoIκBα and α-tubulin. (C) The intensity of bands were densitometrically scanned and normalized with that of α-tubulin in panels (i) and (ii). The values presented are mean ± SEM. *p<0.005 vs. uninduced cells; **p<0.01 vs. TNF-α-induced cells, statistical significance was set at p<0.05. (D) The cells were treated with MG-132 (a proteosome inhibitor; 50 µg/ml) for 30 mins and then exposed to TNF-α (10 ng/ml) for the indicated times. Total cell extracts were prepared and immunoprecipitated with anti-IKK-β antibody followed by kinase assay using GST-IκBα as a substrate. The extracts were also subjected to western blot analysis using anti-IKK-β antibody. (E) The cells were treated with MPTAG and MG-132 and then induced with TNF-α for 30 mins followed by kinase assay and western blot analysis as stated above. (F) The total cell extracts were prepared from cells in absence and presence of TNF-α induction (10 ng/ml) and were immunoprecipitated with anti-IKK-β antibody. The kinase assay was performed in the absence or presence of the indicated concentrations of MPTAG. The extracts were also subjected to western blot analysis using anti-IKK-β antibody.

To determine whether the inhibitory action of MPTAG on NF-κB activation was due to inhibition of IκBα degradation and its phosphorylation, we examined the IκBα and phosphorylated IκBα levels in the cytoplasm. As expected, TNF-α induced the degradation of IκBα within 10 mins, which was concomitant to IκBα phosphorylation (data not shown). Interestingly, MPTAG pretreatment inhibited TNF-α-induced phosphorylation [Figures 4B and 4C *panel (ii)*] and degradation [Figures 4A and 4C *panel (i)*] of IκBα.

Since IKK-β is required for the phosphorylation of IκBα in these cells, we investigated whether MPTAG affected IKK activation. For this, we immunoprecipitated IKK-β from the untreated and MPTAG-treated cells in absence and presence of TNF-α induction and analyzed by western blot. Our results showed that TNF-α induction activated IKK-β within 10 minutes of its addition (Figure 4D) and MPTAG pretreatment appreciably suppressed this activation in a dose-dependent manner (Figure 4E). Interestingly, neither TNF-α nor MPTAG affected the protein levels of IKK-β (Figures 4D and 4E, *lower panels*). To evaluate whether MPTAG suppressed IKK activation directly by binding physically to IKK-β, firstly IKK-β was immunoprecipitated from uninduced and TNF-α-induced cells followed by its incubation with MPTAG and then performing the kinase assay. It was observed that MPTAG did not affect the activity of IKK-β by direct interaction (Figure 4F). Collectively these data suggest that MPTAG blocked the TNF-α-induced activation of IKK-β but neither bound IKK-β directly, nor changed the expression levels of IKK-β.

### MPTAG inhibits TNF-α-induced activation of Akt and its association with IKK-β

**Figure 5:**
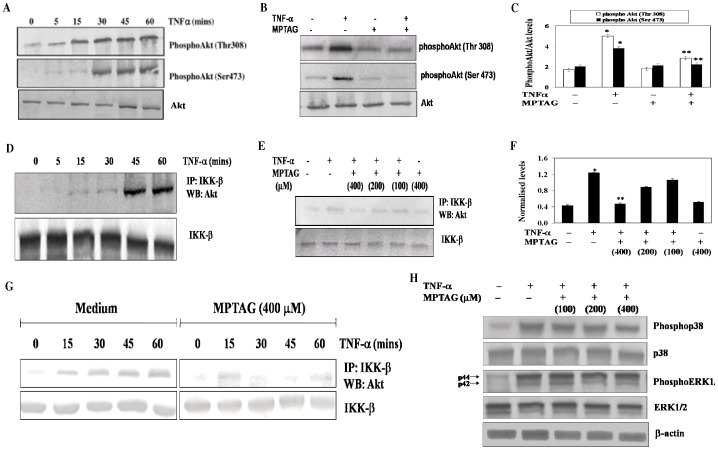
MPTAG inhibits the TNF-α-induced Akt activation and its association with IKK-β. (A) The cells were induced with TNF-α (10 ng/ml) for the indicated times. The total cell extracts were prepared and subjected to western blot analysis using anti-phosphoAkt, for both Ser473 and Thr308 residues, and anti-Akt antibodies. (B) The cells were treated with MPTAG (400 µM) and induced with TNF-α for 30 mins followed by western blot analysis as stated above. (C) The intensity of bands were densitometrically scanned and normalized with total Akt levels. The values presented are mean ± SEM. *p<0.05 vs. uninduced cells; **p<0.05 vs. TNF-α-induced cells, statistical significance was set at p<0.05. (D) The cells were induced with TNF-α (10 ng/ml) for the indicated times. The total cell extracts were prepared and immunoprecipitated with anti-IKK-β antibody followed by western blot analysis with anti-Akt and anti-IKK-β antibodies. (E) The cells were pretreated with MPTAG at varying concentrations and induced with TNF-α for 30 mins. The total cell extracts were prepared and processed as stated above and analyzed for western blot as stated above. (F) The intensity of bands were densitometrically scanned and normalized with IKK-β levels. (G) The cells were pretreated with 400 µM MPTAG and then stimulated with 10 ng/ml TNF-α for the indicated times. The total cell extracts were prepared, immunoprecipitated with anti-IKK-β antibody and analyzed by western blot using anti-Akt and anti-IKK-β antibodies. (H) **Effect of MPTAG on TNF-α-induced p38 MAPK and ERK1/2 activation.** The cells were treated with MPTAG and induced with TNF-α as stated above. The total cell extracts were prepared and analyzed by western blot using anti-phosphospecific p38 MAPK and ERK1/2 antibodies. The same membrane was reblotted with anti-p38 MAPK, ERK 1/2 and β-actin antibodies. The values presented are mean ± SEM. *p<0.05 vs. uninduced cells; **p<0.05 vs. TNF-α-induced cells, statistical significance was set at p<0.05.

Akt is implicated in the regulation of NF-κB activity by activating IKK. Moreover, TNF-α has been shown to activate NF-κB/Rel through the activation of Akt [10], [14]. This indicates the possibility of suppression of TNF-α-induced Akt activation by MPTAG. To examine the effect of MPTAG on activation of Akt by TNF-α, we pretreated cells with MPTAG followed by induction with TNF-α and performed western blot analysis to determine the levels of Akt phosphorylation at both Thr308 and Ser473 residues. The results showed that Akt was phosphorylated in a time-dependent manner at both the residues in TNF-α-stimulated human endothelial cells (Figure 5A). Interestingly, MPTAG inhibited the phosphorylation of Akt at both the residues without affecting the protein levels of Akt (Figures 5B and 5C).

Next we investigated whether MPTAG affected the association of Akt with IKK-β. The results revealed that TNF-α induction caused a time-dependent association of Akt with IKK-β (Figure 5D), and MPTAG significantly prevented this association in a dose-dependent (Figures 5E and 5F) and time-dependent manner (Figure 5G) with no increase in the expression levels of IKK-β in these cells.

As p38 and ERK are also known to be associated with NF-κB activation [13], we tested whether these kinases, are also affected by MPTAG pretreatment. Our results showed that both p38 and ERK were activated in TNF-α stimulated cells but MPTAG pretreatment had no effect on this activation (Figure 5H). These results indicated that MPTAG specifically inhibited the association of Akt with IKK-β in TNF-α stimulated human endothelial cells.

### MPTAG inhibits Akt phosphorylation by a PI-3K independent and PKA-dependent pathway

**Figure 6:**
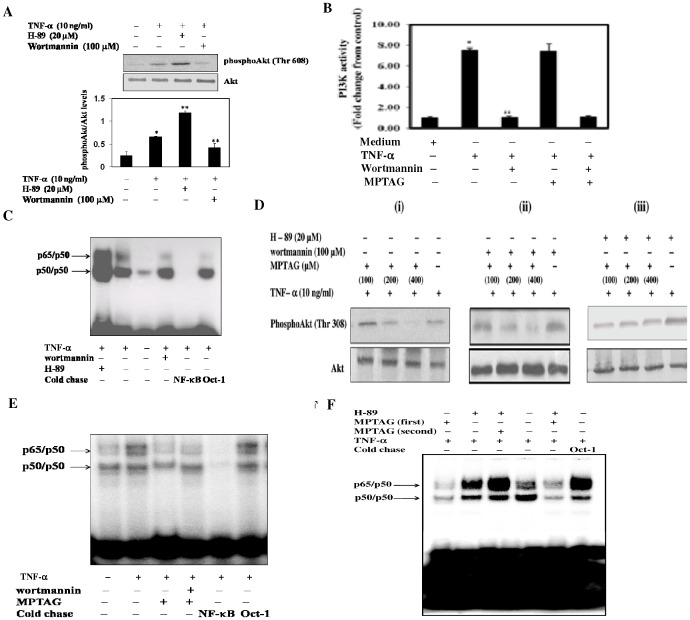
MPTAG blocks Akt activation and NF-κB activation in a PI-3K independent and PKA-dependent manner in TNF-α-stimulated HUVECs. (A) The cells were either preincubated with wortmannin (100 µM) or H-89 (20 µM) for 30 mins and then induced with TNF-α (10 ng/ml) for additional 30 mins. The total cell extracts were subjected to western blot analysis using anti-phospho-Akt (Thr308) and anti-Akt antibodies. (A; lower panel) The intensity of bands were densitometrically scanned and normalized with Akt levels. The values presented are mean ± SEM. *p<0.02 vs. uninduced cells; **p<0.05 vs. TNFα-induced cells, statistical difference was set at p<0.05. (B) The cells were pretreated with wortmannin in absence and presence of MPTAG before induction with TNF-α. The total cell extracts were subjected to PI3K assay (see “Methods”). The mean value (in pmol) for cells treated with neither MPTAG nor TNF-α (control) was set to 1, and -fold changes were determined by comparing values against this set value. The values presented are mean ± SEM. *p<0.002 vs. uninduced cells; **p<0.005 vs. TNF-α-induced cells, statistical significance was set at p<0.05. (C) The cells were treated and induced as described in (A). The nuclear extracts were assessed for NF-κB activation by EMSA. **Effect of MPTAG on Akt phosphorylation in absence and presence of wortmannin and H-89 in TNF-α-stimulated HUVECs.** (D; panels (i–iii)). For this, the cells were incubated without or with either wortmannin or H-89 before treatment with different concentrations of MPTAG followed by induction with TNF-α. The total cell extracts were subjected to western blot analysis using anti-phospho-Akt (Thr308) and anti-Akt antibodies. **Effect of MPTAG on NF-κB activation in absence and presence of wortmannin and H-89 in TNFα-stimulated HUVECs.** (E–F) The cells were treated and induced as stated in (D). The nuclear extracts were prepared and assessed for NF-κB activation by EMSA.

Akt is known to be regulated by upstream kinases PI-3K [14]–[19], [30] and PKA [20]–[24]. To assess the specific roles of these kinases, expressed in human endothelial cells, on Akt phosphorylation, we pretreated these cells with specific inhibitors of PI-3K (wortmannin) and PKA (H-89) before TNF-α induction. We observed that wortmannin inhibited Akt phosphorylation whereas H-89 enhanced it (Figure 6A). Thus, TNF-α-induced Akt phosphorylation was positively regulated by PI-3K and negatively by PKA in HUVECs.

To examine whether MPTAG inhibits Akt phosphorylation by blocking the activity of PI-3K in these cells, the effect of MPTAG with or without wortmannin treatment was assessed. It was observed that MPTAG treatment had no effect on TNF-α-induced PI-3K activity (Figure 6B). To confirm the functional consequence of modulation of Akt phosphorylation, we assessed the NF-κB activation by EMSA. Expectedly, the results showed that wortmannin treatment inhibited and H-89 treatment enhanced the NF-κB activation in TNF-α-stimulated cells (Figure 6C).

Further experiments were then performed to determine the mechanism for the inhibitory effect of MPTAG on TNF-α-induced Akt phosphorylation. The effects of MPTAG on TNF-α-induced Akt phosphorylation was measured in absence and presence of wortmannin or H-89. The results revealed that MPTAG dose-dependently inhibited Akt phosphorylation, independently of wortmannin [Figure 6D *panels (i,ii)*]. Moreover, NF-κB activation, measured by EMSA, was inhibited similarly in presence or absence of wortmannin (Figure 6E), suggesting that the mechanism of action of MPTAG was not through PI-3K inhibition.

In contrast, H-89 added prior to MPTAG showed an interaction where the inhibitory effect of MPTAG was completely abolished by H-89, thereby restoring TNF-α-induced Akt phosphorylation [Figure 6D *panels (i,iii)*]. Further EMSA experiments showed that MPTAG alone significantly inhibited NF-κB activation; H-89 treatment alone enhanced it; when H-89 treatment preceded MPTAG, the activation remained increased; and when MPTAG treatment preceded H-89, the activation was inhibited (Figure 6F).

This strongly suggested that MPTAG and H-89 have opposite actions on PKA-Akt axis. The data best fitted a model where activation of PKA by MPTAG inhibits Akt phosphorylation, thereby inhibiting NF-κB activation, in TNF-α-induced human endothelial cells.

### MPTAG restores PKA activity in TNF-α-stimulated endothelial cells

**Figure 7:**
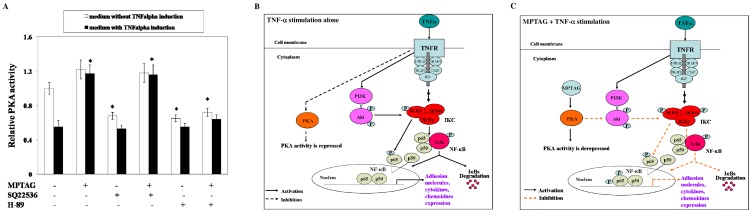
MPTAG restores PKA activity in TNFα-stimulated HUVECs. (A) The cells were treated with media (control), MPTAG, SQ 22536, H-89 or their indicated combinations in absence and presence of TNF-α stimulation. PKA activity was assessed in the cell lysates. Results are expressed as mean ± sem of three independent experiments. *p<0.05 vs. control. **Proposed model of MPTAG action in TNF-α-stimulated HUVECs.** (B) In TNF-α-stimulated HUVECs (without MPTAG treatment), PI-3K-regulated Akt is activated (indicated by solid line arrow) resulting in NF-κB activation. On the other hand, PKA activity remained repressed (indicated by broken line arrow) under these conditions. (C) MPTAG pretreatment to TNF-α-stimulated HUVECs restored the repressed activity of PKA. Thus, derepression of PKA activity resulted in inhibition of Akt and overall inhibition of NF-κB in these cells.

To validate the model, we checked the levels of PKA kinase activity in human endothelial cells. The results, shown in Figure 7A, revealed the presence of appreciable PKA activity in unstimulated HUVECs. Since PKA activation is mostly regulated through the cAMP-dependent pathway involving adenylate cyclase activation, we tested whether MPTAG has any effect on the activity of this enzyme. We observed that PKA activity was moderately suppressed with adenylate cyclase inhibitor SQ 22536 treatment alone but remained increased when MPTAG and SQ 22536 were added together. PKA activity was also found to be significantly repressed upon TNF-α stimulation to these cells. MPTAG added alone or together with SQ 22536 restored PKA activity to levels similar to that of unstimulated cells (Figure 7A). H-89 treatment inhibited PKA activity, even in presence of MPTAG. Thus, restoration of repressed PKA activity by MPTAG is independent of the classical cAMP-dependent regulatory pathway but remains susceptible to catalytic subunit inhibitors like H-89.

## Discussion

Here, we demonstrate for the first time that a novel compound, MPTAG, inhibits NF-κB activation by blocking the phosphorylation of Akt, through a PI-3K independent mechanism, in TNF-α-stimulated HUVECs. As NF-κB is a key regulator of various inflammatory and cellular immune responses and its aberrant expression leads to many pathological conditions, identifying small molecules for regulating NF-κB is an intensive area of research [31]. Over the years, numerous molecules have been identified that blocked the activation of NF-κB by intervening with the signaling through the canonical NF-κB pathway, however, in the present study we provide the first evidence that NF-κB could also be regulated through the PKA/Akt axis in TNF-α-stimulated HUVECs.

The present study identified a novel small molecule MPTAG, a derivative of plant-derived molecule MPG that blocked TNF-α-induced NF-κB activation in HUVECs, through inhibition of Akt activation and subsequent inhibition of Akt-IKK association. An outline of our findings is illustrated in Figures 7B and 7C. Our results revealed that MPTAG inhibited the NF-κB activation through blocking the phosphorylation and degradation of IκBα and nuclear translocation of NF-κBp65 subunit in HUVECs. Since direct incubation of MPTAG with the immunoprecipitated IKK-β showed no effect on its activity, it is likely that MPTAG inhibits NF-κB activation through the suppression of TNF-α-induced IKK-β activation. Akt, also activated by TNF-α, has been shown to associate with and subsequently activate IKK as shown previously in cells other than HUVECs [10], [32]. In this study, we showed that TNF-α stimulation resulted in increased Akt activation and time-dependent association of Akt to IKK-β. In this context, we demonstrated that MPTAG pretreatment inhibited both the TNF-α-induced Akt activation as well as Akt-IKK-β association. Collectively, these results indicated that MPTAG blocked IKK-β activation by suppressing Akt activation in these cells. Most importantly, the lack of effect of MPTAG on TNF-α-induced ERK and p38 MAPK activation suggested specificity of its action on Akt in these cells. Consistent with the previous findings that TNF-α-induced Akt activation augments NF-κBp65 transactivation [29], [33], we showed that p65 phosphorylation increased in a time-dependent manner upon TNF-α stimulation in HUVECs. Interestingly, MPTAG was found to inhibit the TNF-α-induced p65 phosphorylation in these cells. It is important to note that as TNF-α is the early proinflammatory cytokine released from LPS- or phorbol ester-stimulated macrophages and neutrophils [34], [35], MPTAG could also be effective for inhibiting NF-κB activation in inflammatory conditions initiated by these agents.

Further investigations were aimed at elucidating the mechanism through which MPTAG inhibited TNF-α-induced Akt activation in HUVECs. In this context, our results revealed that TNF-α stimulation increased the PI-3K activity in these cells. However, pretreatment of these cells with wortmannin abolished this activation whereas, MPTAG pretreatment had no effect on this activation indicating MPTAG acts independent of conventional PI-3K inhibition in HUVECs. There are numerous reports that showed either positive [20], [21] or negative [22]–[24], [36] regulation of Akt by Protein kinase A (PKA) in various cell types excluding HUVECs. Our study demonstrated that TNF-α stimulation resulted in repression of PKA activity and increased activation of PI-3K and NF-κB pathways in HUVECs. This is in agreement with a report that showed that TNF-α could downregulate PKA activity in podocytes [37]. Interestingly, MPTAG significantly restored the repressed PKA activity in TNF-α-stimulated HUVECs independent of adenylyl cyclase activation. It is probable that restoration of the otherwise repressed PKA activity, by MPTAG, leads to the PI-3K independent inhibition of Akt. We speculate that high constitutive baseline activity of PKA in these cells precludes further activation by MPTAG except when the activity has been repressed by TNF-α, permitting derepression. Interestingly, we observed that MPTAG had no significant inhibitory effect on the basal NF-κB activation, but PKA can still signal at a basal level without TNF-α stimulation.

MPTAG showed improved properties in terms of aqueous solubility, cellular tolerability and inhibitory activity (Tables S1, S2, S3). MPTAG, used at 400 µM, showed complete inhibition of NF-κB activation without affecting the morphology and viability in HUVECs. Here, we would like to mention that this is not as high as the concentrations at which non-steroidal anti-inflammatory molecules like sodium salicylate, aspirin, N-acetyl cysteine, diclofenac inhibit NF-κB [38]–[41] and this study did not intend to propose MPTAG as a unique specific NF-κB inhibitor. Importantly, unlike small molecules that directly inhibit IKK-β, MPTAG only suppressed its activation. This action of MPTAG would contribute towards overcoming the undesirable effects caused by specific IKK-β inhibitors in animal models [42]. We speculate that MPTAG or its improved analogues along with other small molecule IKK inhibitors could provide robust inhibition of NF-κB in severe inflammatory disease conditions where both PKA/Akt axis and IKK pathways are modulated.

## Materials and Methods

### Ethics statement

Human umbilical cords were obtained from St. Stephens Hospital, Delhi, India. A formal ethical clearance certificate, to collect the human cord sample, was obtained from the hospital ethics committee.

### Reagents

D-glucose, red phosphorus, acetic anhydride, HClO_4_, maltol, silver carbonate, dichloromethane, ethyl acetate, petroleum ether were purchased from Qualigens Fine Chemicals (India). M199 powdered medium, L-glutamine, endothelial cell growth factor (ECGF), trypsin, penicillin, streptomycin, amphotericin B, dimethyl sulfoxide (DMSO), MTT, wortmannin, H-89, SQ 22536, GST-IκBα(1–54) substrate, goat anti-mouse IgG conjugated to HRP, mouse IgG conjugated to FITC, goat anti-rabbit IgG conjugated to HRP and Protein G immunoprecipitation kit were purchased from Sigma Chemical Co. (St. Louis, MO). Fetal calf serum (FCS), EZ-RNA isolation kit and EZ-first strand cDNA synthesis kit were purchased from Biological Industries (Kibbutz Beit Haemek, Israel). Antibodies against human ICAM-1, VCAM-1, E-selectin and recombinant human tumor necrosis factor-α (TNF-α) were purchased from BD Pharmingen (San Diego, CA). Primer sets for RT-PCR of human ICAM-1, VCAM-1, E-selectin and β-Actin genes were custom synthesized by Genset Corporation (Tokyo, Japan). NF-κB and Oct-1 oligonucleotides for electrophoretic mobility shift assay (EMSA), Profluor PKA activity and luciferase assay kits were purchased from Promega Inc. (Madison, USA) and Class III PI3-kinase kit was obtained from Echelon (USA). Antibodies against NF-κBp65 (C-20), phospho NF-κBp65, IκBα, phosphoIκBα, IKK-β, Akt, phosphoAkt(Ser473), phosphoAkt(Thr308), PKAα(W-18), p85α(N-18), α-tubulin and Lamin B1 were purchased from Santa Cruz Biotechnology (Santa Cruz, CA).

### Synthesis of MPTAG

Detailed experimental protocol afforded the synthesis of 2-methyl-pyran-4-one-3-O-β-D-2′,3′,4′,6′-tetra-O-acetyl glucopyranoside (MPTAG) (4, Figure S1). In the synthetic scheme, β-D-glucose was treated with HClO_4_, red phosphorous, acetic anhydride followed by water afforded 1-bromo-2,3,4,5-tetra-O-acetyl-β,α-D-glucopyranoside (3) [43] which with maltol (2) in presence of silver carbonate as catalyst produced MPTAG (4) [44]. To a solution of 3-hydroxy-2-methyl-4-pyrone (2; 0.12 g, 0.95 mmol) in dry dichloromethane (50 ml) in 4 Å molecular sieves (1.0 g) was added acetobromoglucose (3; 0.41 g, 1.68 mmol) and silver carbonate (1.4 g, 5 mmol) in succession. The mixture was stirred at room temperature for four days. The suspension was then filtered through small length celite and evaporated to dryness under reduced pressure. The residue was chromatographed over silica gel using petroleum ether and increasing proportion of ethyl acetate. Petroleum ether: ethyl acetate eluent (7∶3) afforded pure MPTAG (4, Figure S1) as colorless sticky solid (0.278 g, 61%).

### 2-methyl-pyran-4-one-3-O-β-D-2′,3′,4′,6′-tetra-O-acetylglucopyranoside [MPTAG (4)]

IR: ν_max_ (KBr, cm^−1^): 2924, 2847, 1762, 1372, 1212; ^1^H-NMR (300 MHz, DMSO-d_6_) : δ 1.91 (s, 3H), 1.97 (s, 3H), 1.98 (s, 3H), 2.04 (s, 3H), 2.26(s, 3H), 3.96–4.15 (m, 3H), 4.91–4.99 (m, 2H), 5.23 (d, J = 7.8 Hz, 1H), 5.32–5.38 (m, 1H), 6.37 (d, J = 5.6 Hz, 1H), 8.07 (d, J = 5.6 Hz, 1H); ^13^C-NMR (75 MHz, DMSO-d_6_): δ 15.77, 21.12, 21.24 (2C), 21.37, 62.25, 68.96, 71.48, 71.94, 72.48, 100.28, 117.42, 141.88, 156.25, 161.93, 170.22 (2C), 170.39, 170.81, 173.64 ESI MS m/z: 478.92 [M+Na]^+^; Anal. Calcd for C_20_H_24_O_12_: C, 52.63; H, 5.30. Found: C, 52.34; H, 5.19.

### Human umbilical vein endothelial cells (HUVECs) culture

Primary endothelial cells were isolated from human umbilical cord as described previously [28]. The cells were grown in M199 medium supplemented with 15% heat inactivated fetal calf serum, 2 mM L-glutamine, 100 units/ml penicillin, 100 µg/ml streptomycin, 0.25 µg/ml amphotericin B, and endothelial cell growth factor (ECGF; 50 µg/ml).

### Preparation of stock solutions of MPG and its derivatives

Stock solutions of MPG and its derivatives (Tables S1, S2, S3) were prepared either in dimethylsulphoxide (DMSO) or cell culture medium. MPG and its derivatives were diluted to working concentrations in cell culture medium. The highest concentration of DMSO in control wells was 0.25% which did not alter the morphology, viability of the cells and had no effect on ICAM-1 assay (data not shown).

### Cytotoxicity assay

The cytotoxicity of MPG and its derivatives was analyzed by colorimetric assay using MTT (Thiazolyl blue tetrazolium bromide) reagent as described previously [28]. All experiments were performed at least 3 times in triplicate wells. From this assay, the percentage viability (% viability) of the cells at various concentrations of each compound was determined by normalization to control wells that contained cells incubated in vehicle (0.25% DMSO or cell culture medium) and which were considered 100% viable. The highest concentration at which the viability of the cells was >95% was denoted as the maximum tolerable concentration for that compound.

### Cell-ELISA for measurement of ICAM-1, VCAM-1 and E-selectin expression

The endothelial cells were incubated with or without MPG and its derivatives at various concentrations for 2 h followed by treatment with TNF-α (10 ng/ml) for 16 h for ICAM-1 and VCAM-1 expression and 4 h for E-selectin expression. A Cell-ELISA was used to assess the expression of ICAM-1, VCAM-1 and E-selectin on the surface of endothelial cells as described previously [28].

### Neutrophil adhesion assay

Neutrophils were isolated from peripheral blood of healthy individuals and neutrophil adhesion assay was performed under static conditions as described previously [28], [45].

### Preparation of cytoplasmic and nuclear extracts

The endothelial cells were subjected to the indicated treatments followed by induction with TNF-α for indicated time points or 30 mins. The cells were then washed with PBS and dislodged using a cell scraper. The cytoplasmic and nuclear extracts were prepared as described previously [28], [46].

### Total RNA isolation and Reverse Transcription-Polymerase Chain Reaction (RT-PCR)

In order to examine whether MPTAG reduced the mRNA transcript levels of cell adhesion molecules genes, the endothelial cells were pretreated with 400 µM MPTAG for 2 h before induction with TNF-α for 4 h followed by RT-PCR. RNA was isolated from MPTAG-treated cells according to a modified guanidinium thiocyanate procedure. The expression of the transcripts for ICAM-1, VCAM-1 and E-selectin was evaluated by RT-PCR [45]. β-Actin levels expressed under similar conditions were used as a loading control.

### Nuclear transcription factor-κB (NF-κB) activation assay

NF-κB activation in the nuclear extracts of human endothelial cells was assessed by electrophoretic mobility shift assay (EMSA) as described previously [45], [46]. For supershift experiment, nuclear extracts prepared from unstimulated and TNF-α-stimulated cells were incubated with antibodies against the p50 or p65 subunit of NF-κB or -α-tubulin for 15 mins at 37°C before the complex was analyzed by EMSA. Addition of equal amount of radioactive-labelled probe to all the samples was indicated by the similar intensity of the unincorporated probe observed towards the end of the gel.

### NF-κB reporter gene assay

NF-κB dependent reporter gene transcription was measured as per the instructions of the luciferase assay kit (Promega, USA). Briefly, human endothelial cells (2×10^6^ cells/well) were plated in 96-well plates. HUVECs were transiently transfected by electroporation with a NF-κB-containing luciferase reporter gene plasmid for 24 h. After transfection, cells were washed and treated with 400 µM MPTAG for 24 h. For the TNF-α stimulation, cells were treated with 10 ng/ml of TNF-α for another 24 h. Supernatants after cell lysis were assayed for luciferase activity. The mean value for cells treated with no MPTAG and no TNF-α was set to 1, and -fold differences were determined by comparing values against this set value. Cells were co-transfected with a control vector expressing β-galactosidase under SV40 promoter to normalize transfection efficiency.

### Immunochemical staining of HUVECs

To study the effect of MPTAG on nuclear translocation of NF-κBp65 subunit in presence of TNF-α-stimulation, the HUVECs were seeded on gelatinized glass bottom 6-well plate at a cell density of 0.3×10^6^ cells/ml. Following day, media was changed and MPTAG was added to the cells for 2 h. After the end of drug pretreatment, cells were induced with TNF-α for 45 mins. The cell monolayers were washed with PBS (phosphate buffered saline containing 1 mM CaCl_2_ and 1 mM MgCl_2_) and were fixed in a solution, containing 4% paraformaldehyde in 120 mM sodium phosphate buffer pH 7.2–7.4, for 20 mins at room temperature. The cells were then permeabilized using a high salt buffer, containing 0.45 M NaCl and 20 mM sodium phosphate buffer pH 7.2–7.4, 0.3% Triton X-100 and 0.2% gelatin, for 1 h at room temperature. Following this, the cells were incubated with anti-NF-κBp65 antibody for 2 h at room temperature. The excess antibody was washed five times with high salt buffer to ensure removal of unbound antibody. This was followed by incubation with anti-rabbit FITC-labeled secondary antibody for 1 h at room temperature. The cells were washed five times with high salt buffer and the final wash with PBS containing 1 mM CaCl_2_ and 1 mM MgCl_2_. The cells were mounted using a mounting medium containing DAPI and anti-fade agent. The cells were observed under a Nikon fluorescent microscope. The fluorescent signals were then quantitated by selecting the nuclear and cytoplasmic regions in different fields using the NTS imaging software.

### Immunoprecipitation protocol

In brief, confluent HUVECs were incubated with or without MPTAG and were stimulated with TNF-α (10 ng/ml) for 15 mins. The cells were washed and lysed in Lysis buffer containing 50 mM HEPES, pH 7.6, 10% glycerol, 1 mM sodium orthovanadate, 100 mM NaCl, 1% NP-40, 1 mM EDTA and protease inhibitor cocktail and allowed to swell on ice for 45 mins. Following centrifugation at 13,000 rpm for 45 mins, the supernatants were collected as “total cell extracts” and stored at −70°C. Immunoprecipitation was performed as per the instructions of Protein G immunoprecipitation kit (Sigma Aldrich, USA). Endogenous IKK complexes were immunoprecipitated in the following reaction mixtures: 100–300 µg of total cell extracts were incubated with 2–4 µg anti-IKK-β or PKAα antibody in 100 µl 1× IP buffer. After incubation for 16 h at 4°C with constant shaking, 50 µl of activated protein G beads were added and the mixture was incubated under rotation for an additional 3 h at 4°C.

### IKK-β kinase assay

Kinase assay was performed as previously described [32]. Immunoprecipitates from 100 µg of total cell extracts were used for kinase assays. The reaction mixture consisted of kinase buffer (20 mM Tris Cl, pH 7.6, 1 mM EDTA, 1 mM sodium orthovanadate, 20 mM MgCl_2_, 2 mM DTT), 2 µg GST-IκBα(1–54), 5 µM ATP and 1 µCi [γ ^32^P] ATP in a volume of 30 µl. Kinase reactions were performed at 37°C for 30 mins and then subjected to SDS-PAGE and autoradiography.

### Western blot analysis

Nuclear and cytoplasmic extracts were fractionated on SDS-PAGE with equal amount of proteins per well and transferred onto PVDF membranes and those membranes were incubated with appropriate primary antibodies and treated with HRP conjugated secondary antibodies followed by detection with DAB-H_2_O_2_ system. β-tubulin, α-actin and Lamin B1 were used as loading controls. Antibodies against NF-κBp65, phosphoNF-κBp65, IκBα, phosphoIκBα, IKK-β, Akt, phosphoAkt (Ser473) and phosphoAkt (Thr308) were used.

### Protein kinase A (PKA) activity assay

PKA was immunoprecipitated from the total cell lysates of unstimulated and TNF-α-stimulated endothelial cells in presence and absence of treatment with agents indicated in Figure 7 using anti-PKAα (W-18) antibody. The immunoprecipates were subjected to kinase activity assay using a Bisamide Rhodamine 110 peptide substrate as per the instructions of the Profluor PKA assay kit (Promega, USA).

### Phosphoinositide-3-kinase (PI-3K) activity assay

The catalytic domain of PI-3K was immunoprecipitated from the stimulated and unstimulated endothelial in presence and absence of various compound treatment using an anti-p85α (N-18) antibody. The immunoprecipated proteins were subjected to kinase activity assay using a PI substrate, diC_8_ followed by detection of the PI(3)P as per the instructions of the Class III PI-3kinase kit (Echelon, USA).

### Statistical analysis

Data are expressed as means ± standard error of the mean (s.e.m). For comparisons between two selected groups, we used unpaired student's ‘t’ test. For comparisons between multiple groups, ANOVA with Bonferroni's correction was used. A value of p<0.05 was considered statistically significant. Analyses were done using JMP (version 4.0) and GraphPad Prism (version 5.0) softwares.

## Supporting Information

Figure S1 **Scheme of synthesis of 2-methyl-pyran-4-one-3-O-β-D-2′,3′,4′,6′-tetra-O-acetyl glucopyranoside (MPTAG).** In the synthetic scheme, β-D-glucose was treated with HClO4, red phosphorous, acetic anhydride followed by the water afforded 1-bromo-2,3,4,5-tetra-*O*-acetyl-β,α-D-glucopyranoside (3) which with maltol (2) in presence of silver carbonate (Ag_2_CO_3_) and CH_2_Cl_2_ as catalyst gave the MPTAG (4).
(TIF)

Table S1 **Structures of derivatives of 2-methyl-pyran-4-one-3-O-β-D-glucopyranoside (MPG).** The derivatives of parent compound MPG were synthesized in the laboratory and their structures were determined by spectroscopic methods.
(TIF)

Table S2 **Cytotoxicity profiles and ICAM-1 inhibitory activities of the parent compound (MPG) and its derivatives.** The data are expressed as mean ± s.e.m. The results are representative of three independent experiments.
(TIF)

Table S3 **The inhibitory profile of MPTAG vs. MPG on human endothelial cells.** The data are expressed as mean ± s.e.m. The results are representative of three independent experiments.
(TIF)
