# Supplementary figures and images for: Regulation of NF-κB Activation through a Novel PI-3K-Independent and PKA/Akt-Dependent Pathway in Human Umbilical Vein Endothelial Cells
Source: PLoS One. 2012 Oct 5;7(10):e46528. doi: 10.1371/journal.pone.0046528 (PMC3465347; doi:10.1371/journal.pone.0046528)

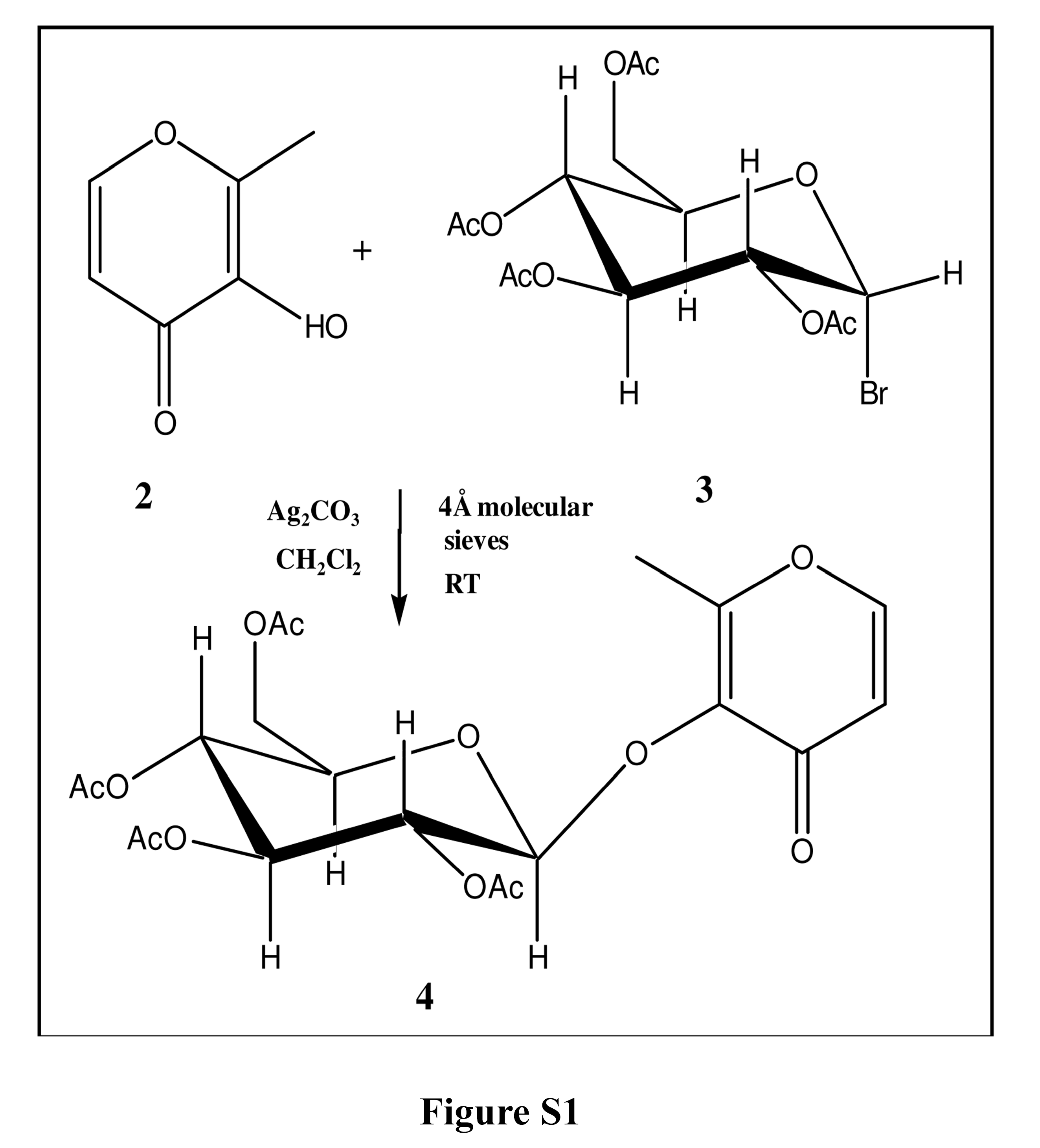

Supplement: Figure S1 — Scheme of synthesis of 2-methyl-pyran-4-one-3-O-β-D-2′,3′,4′,6′-tetra-O-acetyl glucopyranoside (MPTAG). In the synthetic scheme, β-D-glucose was treated with HClO4, red phosphorous, acetic anhydride followed by the water afforded 1-bromo-2,3,4,5-tetra-O-acetyl-β,α-D-glucopyranoside (3) which with maltol (2) in presence of silver carbonate (Ag2CO3) and CH2Cl2 as catalyst gave the MPTAG (4). (TIF) [file pone.0046528.s001.tif]

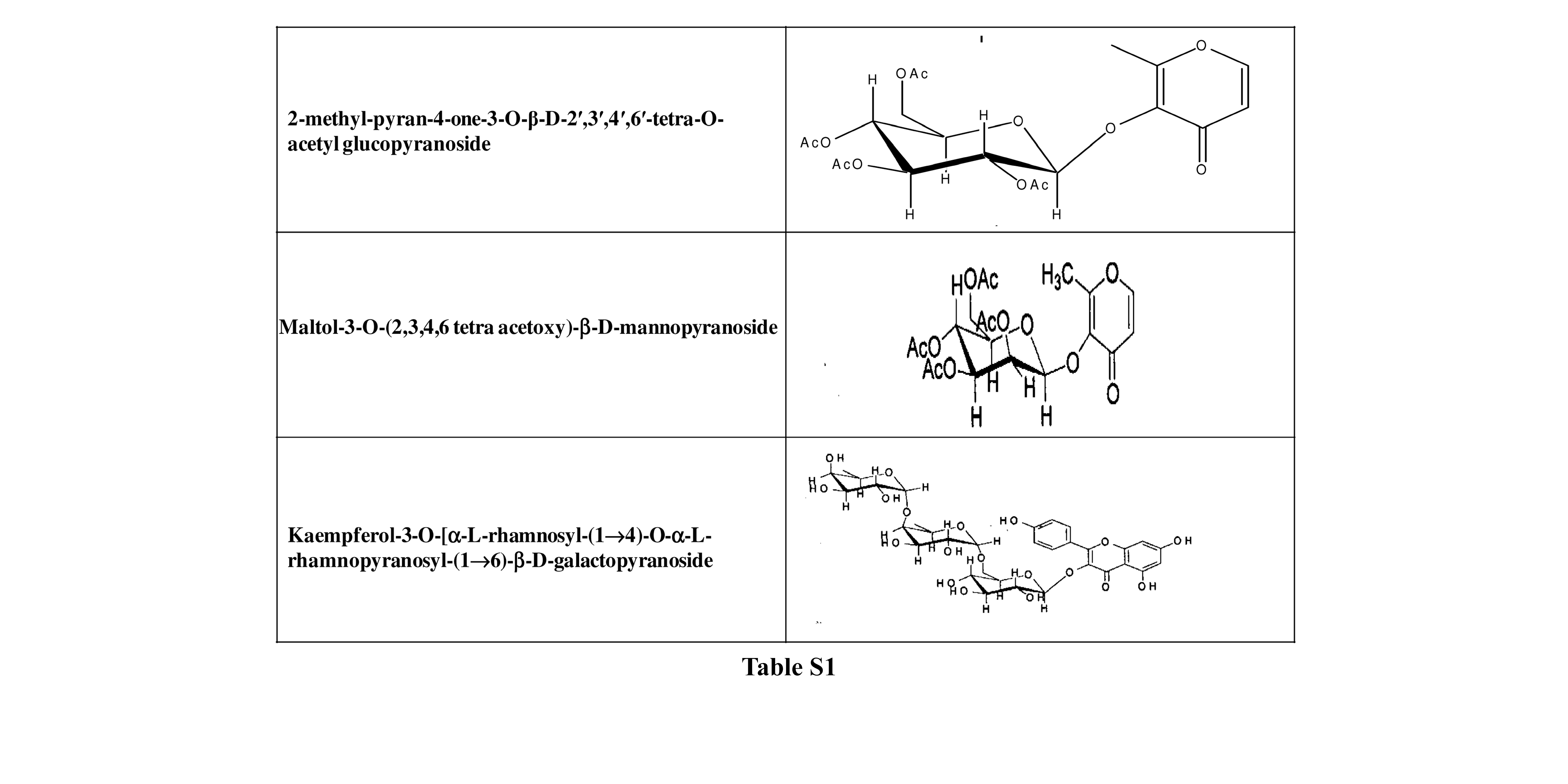

Supplement: Table S1 — Structures of derivatives of 2-methyl-pyran-4-one-3-O-β-D-glucopyranoside (MPG). The derivatives of parent compound MPG were synthesized in the laboratory and their structures were determined by spectroscopic methods. (TIF) [file pone.0046528.s002.tif]

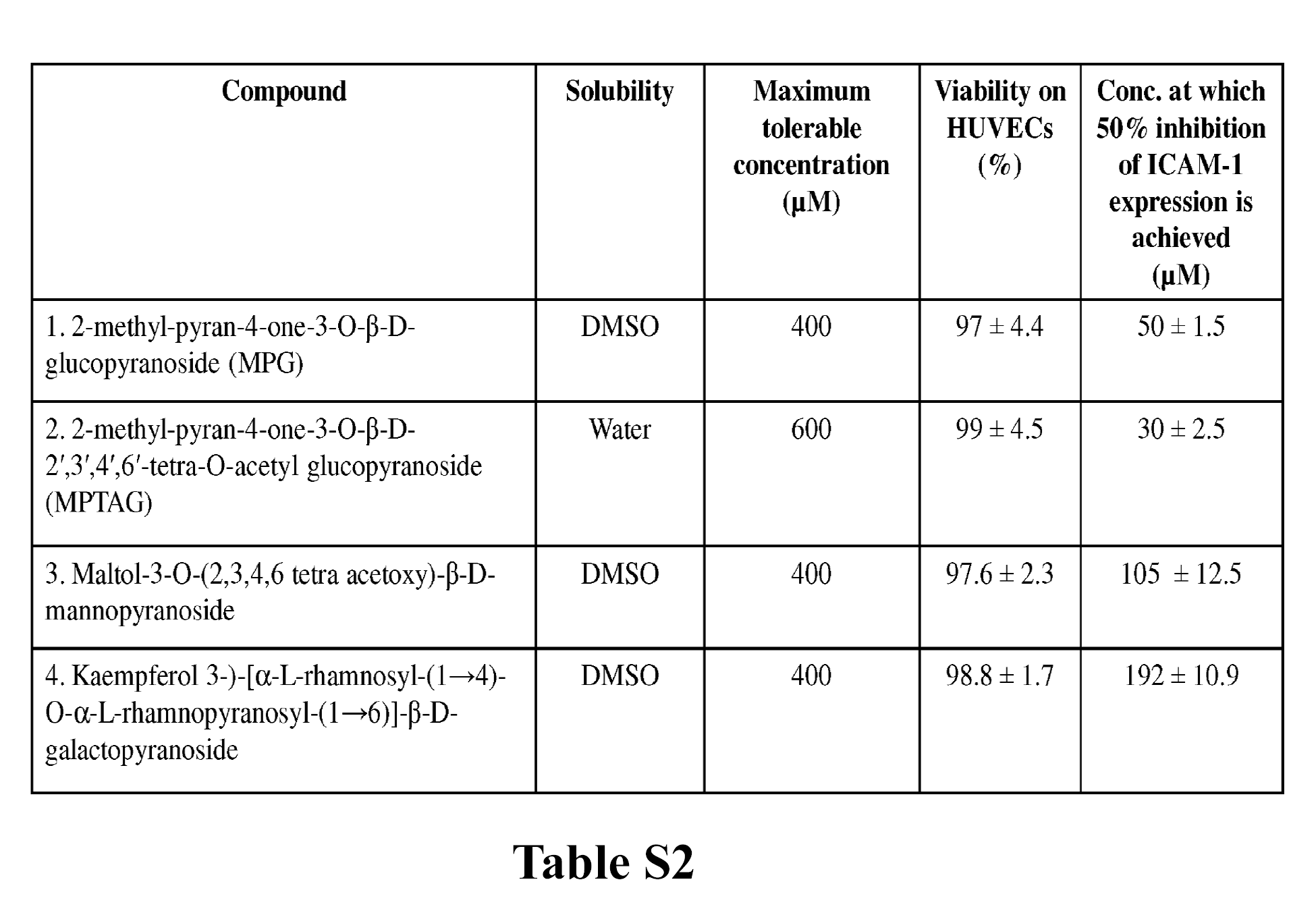

Supplement: Table S2 — Cytotoxicity profiles and ICAM-1 inhibitory activities of the parent compound (MPG) and its derivatives. The data are expressed as mean ± s.e.m. The results are representative of three independent experiments. (TIF) [file pone.0046528.s003.tif]

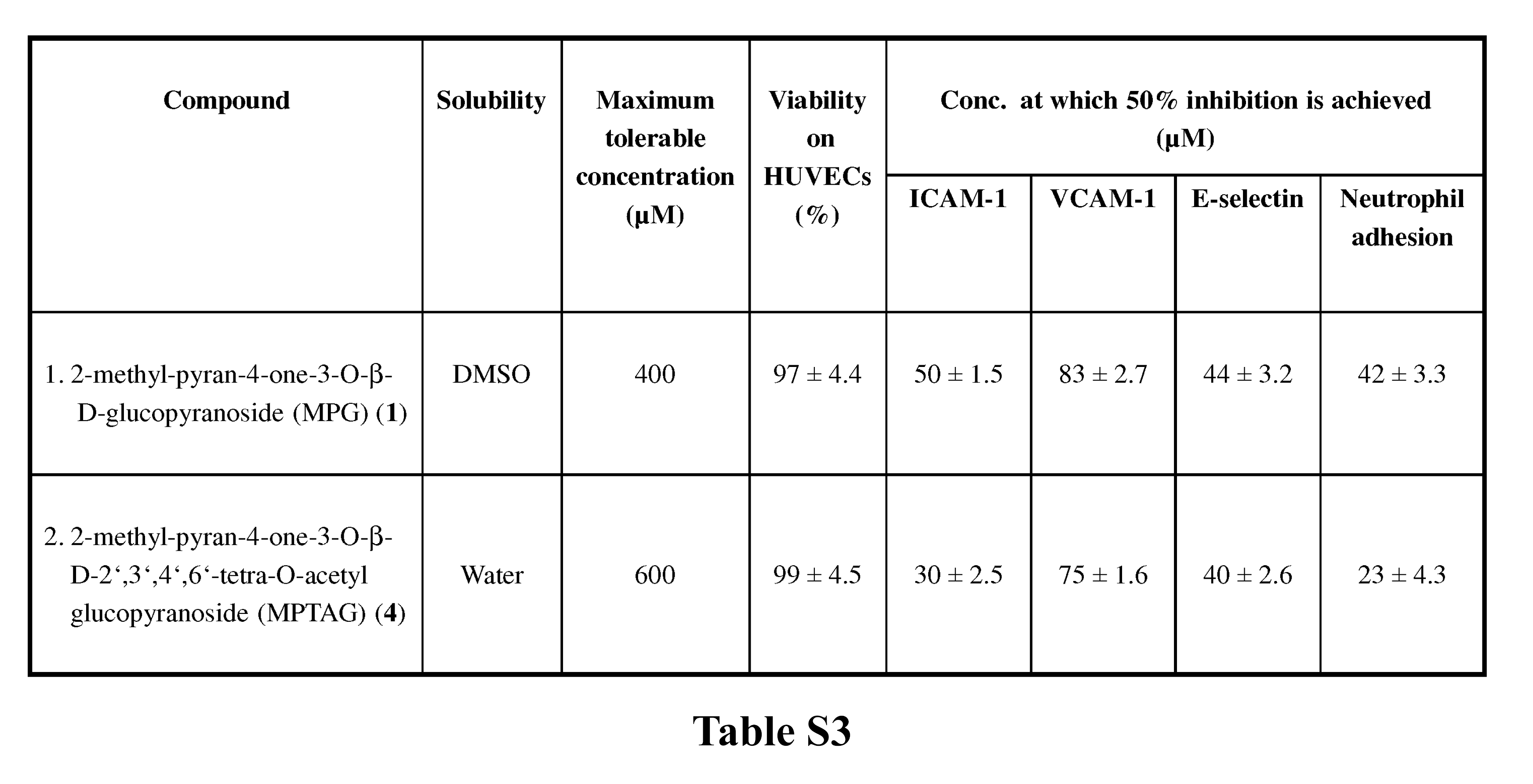

Supplement: Table S3 — The inhibitory profile of MPTAG vs. MPG on human endothelial cells. The data are expressed as mean ± s.e.m. The results are representative of three independent experiments. (TIF) [file pone.0046528.s004.tif]
